# Supplementary material for: Limited thermal tolerance in tropical insects and its genomic signature
Source: Nature. 2026 Mar 4;651(8106):672–8. doi: 10.1038/s41586-026-10155-w (PMC12999521; doi:10.1038/s41586-026-10155-w)
Supplement: Supplementary file 1 — Supplementary Methods: text file including robustness analyses for (i) phylogenetic analyses including Table S1, and (ii) estimations of future climate including Figure S1 [file 41586_2026_10155_MOESM1_ESM.docx]

**Supplementary Methods**

***I. Phylogenetic analyses***

We provide here a set of robustness analyses (test scenarios 1-6) concerning the influence of singleton species, taxonomic assignments, sampling completeness, and modifications of phylogenetic analyses on resulting patterns. For all scenarios, we run ancestral state reconstruction of CT_max_ and checked the values at the nodes representing taxonomic orders (Hymenoptera, Diptera, Lepidoptera, Coleoptera, Hemiptera, Orthoptera) and taxonomic families (Braconidae, Chrysomelidae, Formicidae, Ichneumonidae, Muscidae). Furthermore, for four scenarios we estimated the phylogenetic signal (Pagel’s λ, Blomberg’s k) in predictive models of thermal tolerance and the ϴ parameter of Ornstein-Uhlenbeck models assuming upper boundaries in the evolution of CT_max_. The mean values and standard deviations of these test scenario analyses are presented in Table S1 and compared to values of the original data set presented in the main text. For three test scenarios, in which analyses were conducted with data subsets, we estimated trends in CT_max_ and CT_min_ with elevation and compared these with trends of the full data set (Extended Data Fig. 3).

Scenarios tested:

**1. Species represented by single individuals**

The sampling design of our study focused on covering a very broad range of insect taxa, such that many species (OTU) are only represented by single individuals. In scenario 2, we tested if trends in thermal tolerance with elevation, ancestral state reconstruction and estimates of phylogenetic model parameters are influenced by an excessive number of these singleton species. For robustness testing, we run all analyses with a dataset excluding all singleton species. Excluding singleton species did not lead to significant changes in the reconstruction of ancestral trait states nor in elevational trends of thermal limits. Excluding single OTUs lead to a stronger phylogenetic signal within the CT_max_ data (Table S1).

**2. Potentially wrong family level assignment**

We constructed a phylogenetic tree by calculating individual phylogenetic trees for the species belonging to one taxonomic family and fusing them to a published phylogenetic family-level backbone tree. For this, all DNA barcoded specimens had to be assigned to a taxonomic family. Because morphological assignments, even by experts, repeatedly fail to be correct when it comes to the identification of insects in hyperdiverse tropical environments, where many species also remain unknown, we used DNA barcoding sequences to allocate specimens to taxonomic families. As DNA-sequenced based identification of families may also be error prone, we evaluated the congruence between morphological family assignments (where a confident ID was available) and those obtained through COI barcode matching. We found a high level of agreement: For 89% (1477 matches) of all identified specimens in East Africa and for 91% (1174 matches) in the Neotropics a perfect match was found. Additionally, we run all analyses with a subset of data for which morphological family level assignments were available. The trend of CT_max_ with elevation remained stable, the phylogenetic signal was still significant while slightly stronger, and the evolutionary optimum in CT_max_ was very similar to estimates based on the full data set (Table S1).

**3. Families missing in backbone tree**

The Rainford et al. (2014) insect phylogeny is considered as the most complete, in terms of the number of included families, backbone tree currently existing. However, fourteen samples from our dataset from twelve families (from a total of 242 families) were not included in the backbone tree. These missing families (before the dash) were added manually by placing them next to a sister family (after the dash) included in the backbone:

Ripipterygidae - Tridactylidae

Ischnorhinidae - Clastopteridae

Cyrtocoridae - Cydnidae

Neriidae - Micropezidae

Lyciscidae - Leucospidae

Diparidae - Tetracampidae

Sparasionidae - Scelionidae

Photinaidae - Liturgusidae

Lonchodidae - Phasmatidae

Curtonotidae - Drosophilidae

Chroicopteridae - Metallyticidae

Cybocephalidae - Sphindidae

To test if this addition influenced reconstruction of ancestral states of CT_max_, in a fourth robustness test scenario, we removed these manually added families. The exclusion of the families did not have strong effects on ancestral states of CT_max_ (Table S1).

**4. Non-monophyletic families**

Taxonomy and phylogenetic analysis is a dynamic field with constant changes, and some insect families which are assumed to be monophyletic in the phylogeny of Rainford et al. (2014) turned out to be paraphyletic in more recent phylogenomic studies of specific insect orders. We checked the new phylogenomic studies on Hymenoptera (Blaimer et al., 2023), Coleoptera (Cai et al., 2022), Lepidoptera (Kawahara et al., 2023), Orthoptera (Song et al., 2020), and Hemiptera (Song et al., 2024) for potential cases of paraphyletic families and found, across the whole data set, five families which are probably non-monophyletic. In the robustness analyses of test scenario 5 we excluded all individuals from these potentially non-monophyletic families for data analyses:

Diapriidae, 9 individuals

Elateridae, 22 individuals

Eupelmidae, 2 individuals

Pteromalidae, 7 individuals

Scarabaeidae, 116 individuals

We found that the removal of non-monophyletic families had no impact on any of the results (Table S1).

**5. Limited sampling of taxa**

Reconstruction of ancestral states of traits for a given clade depends on the completeness of taxonomic species sampling, with most precise estimates of ancestral trait states with completely sampled species in a clade. However, since our study comprises a very broad taxonomic range (i.e. insects) with a very high taxonomic diversity, it was impossible for us to completely cover all species nor families of insects. We tested for the influence of sampling completeness by randomly removing 50% of all species (OTUs) from the phylogenetic tree with 100 repetitions. We checked if the reconstructed ancestral states at the above-mentioned nodes (representing insect orders and families) remained stable. Removing half of the data did not have strong effects on estimates of thermal traits for nodes at the level of orders (Table S1).

**6. Uncertain branch lengths**

In the phylogenetic tree, nodes were based on fossil calibration points while unknown ages were evenly distributed between known nodes (bladj function, see Methods). This may introduce imprecise branch lengths. To assess the potential effect of branch lengths on ancestral state reconstruction, we generated branch length perturbations using a set of trees with jittered branch length (N = 50, with a 10% perturbation factor). From these replicates, we calculated mean estimates and standard deviations of ancestral states of CT_max_. Means of ancestral states were stable across the ensemble and standard deviations were low such that our conclusions were not affected (Table S1).

To sum up, all six robustness analyses did not lead to major changes in estimations of elevational trends of CT_max_, of ancestral states of CT_max_, of the θ parameter of OU models nor led to a reduction in the phylogenetic signal of thermal tolerance, underscoring the results reported in the main text.

**Table S1 |** **Robustness tests.** Results of a set of robustness analyses concerning modifications of phylogenetic analyses, considering various scenarios of data subsetting (1–6 as described in SI). (a) Shown are mean values of ancestral state reconstruction of critical thermal maxima (CT_max_) and the absolute differences (ΔCT_max_) of data subsets related to different test scenarios. Values are means ± SD calculated for 6 insect orders and 5 family nodes in the phylogenetic tree. (b) Estimates of phylogenetic parameters Pagel’s λ, Blomberg’s K and the Theta ϴ parameter (estimating the thermal boundary of CT_max_ of OU models) for data subsets testing the robustness of phylogenetic analysis and ancestral trait reconstruction. The column Differences shows the differences of estimated phylogenetic parameters of data subsets to the estimates of the full data set.


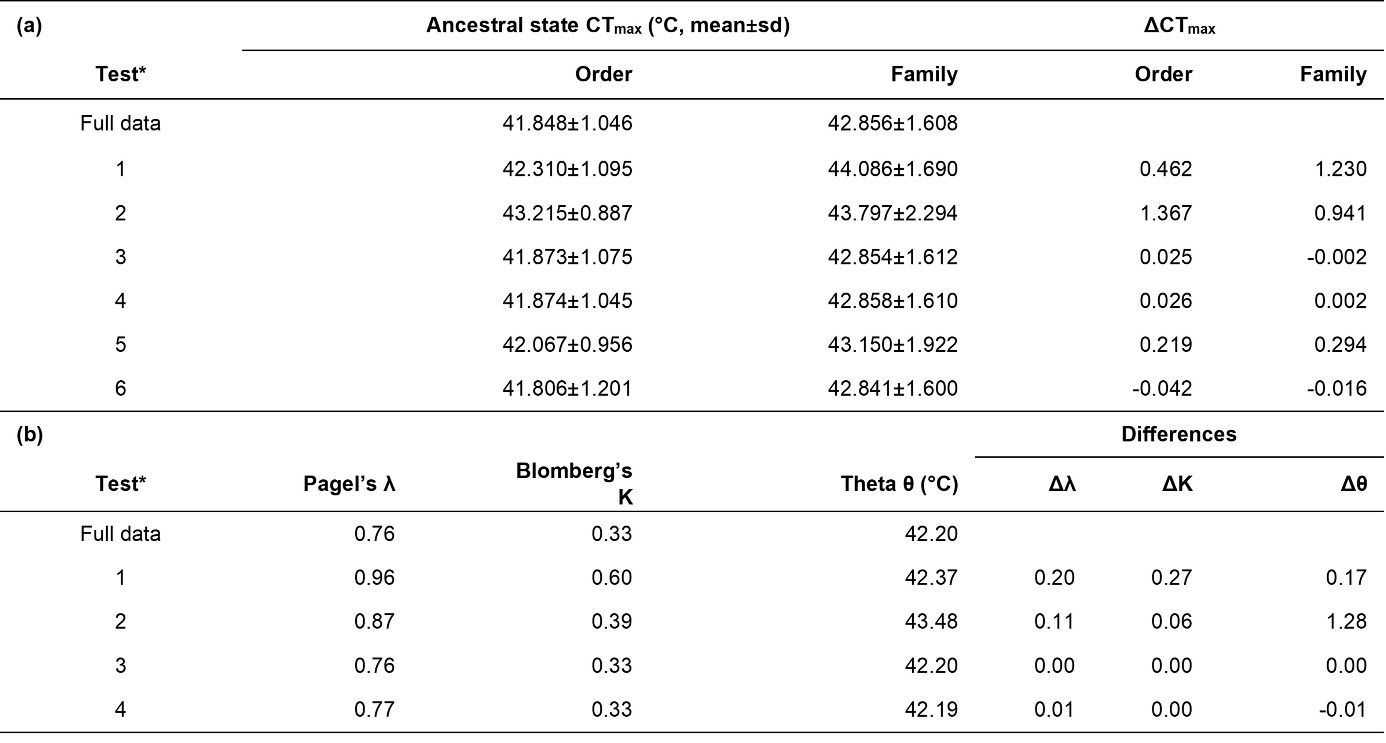
*Test: 1 = species, i.e. OTUs, represented by single individuals were excluded; 2 = only families with a matching morphological and barcode identification were included; 3 = families that were manually added to the backbone tree were excluded; 4 = families considered as non-monophyletic in recent phylogenies were excluded; 5 = random removal of 50% data in 100 repetitions to control for limited sampling; 6 = 10% perturbation in branch lengths to control for uncertain node ages.

***II. Estimations of future climate***

**1. Baseline data**

For climate change projections, we used current microclimate data and added future anomalies (bio5) derived from CHELSA for modelling future microclimatic variation under various climate change scenarios. In a first step, we calculated the differences of microclimatic temperatures to CHELSA’s bio5 variable. The bio5 variable was calculated from historic weather station data of the years 1981–2010, but our microclimate data from field-measurements were collected during 2022–2023. So, our approach assumes that the climatic data of 1981-2010 is representative of the climate at sampling time. Please note that no other climate data sets were suitable for the purpose of our study, as they are either not in a suitable resolution for steep elevational gradients (often >>1 km), or do not cover the time period of our sampling. By calculating bio1 (mean annual temperature) and bio5 (mean maximum temperature of the warmest month) from microclimatic data of data loggers on our study site and comparing these estimates to those calculated CHELSA’s bio1 and bio5 values, we checked if the latter are representative for the current climate. We found a highly significant correlation of the current, field-measured air temperatures to both the mean annual temperature (bio1) from CHELSA (r = 0.994, p < 0.001; Extended Data Fig. 9a) and to the mean warmest month temperature (bio5) from CHELSA (r = 0.962, p < 0.001; Extended Data Fig. 9b). We conclude that the CHELSA bio1 and bio5 variables provide a robust baseline for future climate calculations.

**2. Climate change models**

Climate change projections were calculated for three common shared socio-economic pathways: (1) SSP1-2.6, which belongs to the “Sustainability” pathway, focusing on low consumption, low population growth and effective international collaborations, and ~1.8 °C global warming; (2) SSP3-7.0, part of the “Regional rivalry”, characterized by a security-focused policy with high inequality, and warming of ~3.6 °C; and (3) SSP5-8.5, a worst-case scenario of the “Fossil-fuel development” with high consumption, reduced inequality and low population growth, and ~4.4 °C warming. The numbers behind the SSP indicate the radiative forcing levels in watts per m^2^ expected by the year 2100 (IPCC, 2021).

In the main paper, climate change projections were based on the GFDL-ESM4 model. For consideration of inter-model variability, we have additionally included an ensemble across all available models from CHELSA by extracting the bio5 data projected for 2071-2100 for the three SSPs also from the other four available models (IPSL-CM6A-LR, MPI-ESM1-2-HR, MRI-ESM2-0 and UKESM1-0-LL). Next, we calculated the anomaly for each model by calculating the difference to the historic CHELSA bio5 (Δbio5) and in a final step, we calculated the mean Δbio5 across the five climate change models resulting in a multi-model ensemble estimate of Δbio5. For heat coma time calculations, we estimated future temperatures by adding these multi-model Δbio5 to the current environmental temperatures measured in the field (Neotropics) and modelled microclimate (Afrotropics), as well as for the surface temperatures (ECOSTRESS) on each plot.

Considering climate change anomalies based on a multi-model average instead of using the GFDL-ESM4 alone, the proportion of critical temperatures in the Amazonian lowlands were estimated to be slightly lower (Figure S1): 15% of future surface temperatures under SSP1-2.6 are predicted to cause heat coma in half the insect community within 8 h, 30% under SSP3-7.0, and 36% under SSP 5-8.5 (in comparison to 20%, 39% and 52% based on GFDL-ESM4). For more heat-sensitive insects (25% quantile) the proportion sums up to 17%, 33% and 40% under SSP1-2.6, SSP3-7.0 and SSP 5-8.5, respectively (26%, 45% and 59% under GFDL-ESM4). For the most sensitive insects (10% quantile) the proportion of critical surface temperatures increases to 24%, 36% and 46% under SSP1-2.6, SSP3-7.0 and SSP 5-8.5, respectively (29%, 52% and 67% under GFDL-ESM4). Air temperatures also remain critical under all SSP considering a multi-model ensemble. For the median community, the proportion sums up to 8%, 21% and 28% under SSP1-2.6, SSP3-7.0 and SSP 5-8.5, respectively (9%, 30% and 38% under GFDL-ESM4). For the more heat-sensitive insects, the proportion corresponds to 12%, 26% and 35% under SSP1-2.6, SSP3-7.0 and SSP 5-8.5, respectively (15%, 37% and 47% under GFDL-ESM4). For the most sensitive insects, 24%, 38% and 44% of air temperatures are predicted to be high enough to cause heat coma (under a exposition time of 8h) under SSP1-2.6, SSP3-7.0 and SSP 5-8.5, respectively (25%, 44% and 53% under GFDL-ESM4).

In East Africa, the climate change anomalies calculated using GFDL-ESM4 and those from the multi-model approach were nearly identical, differing only at the first or second decimal place (Figure S1). The GFDL-ESM4 model is known for its focus on carbon cycle feedback, which is likely to play a major role in Amazonian rainforests and explains why this model predicts slightly stronger temperature increases for Neotropical lowlands than the other models (Cano et al., 2022; Parry et al., 2022).


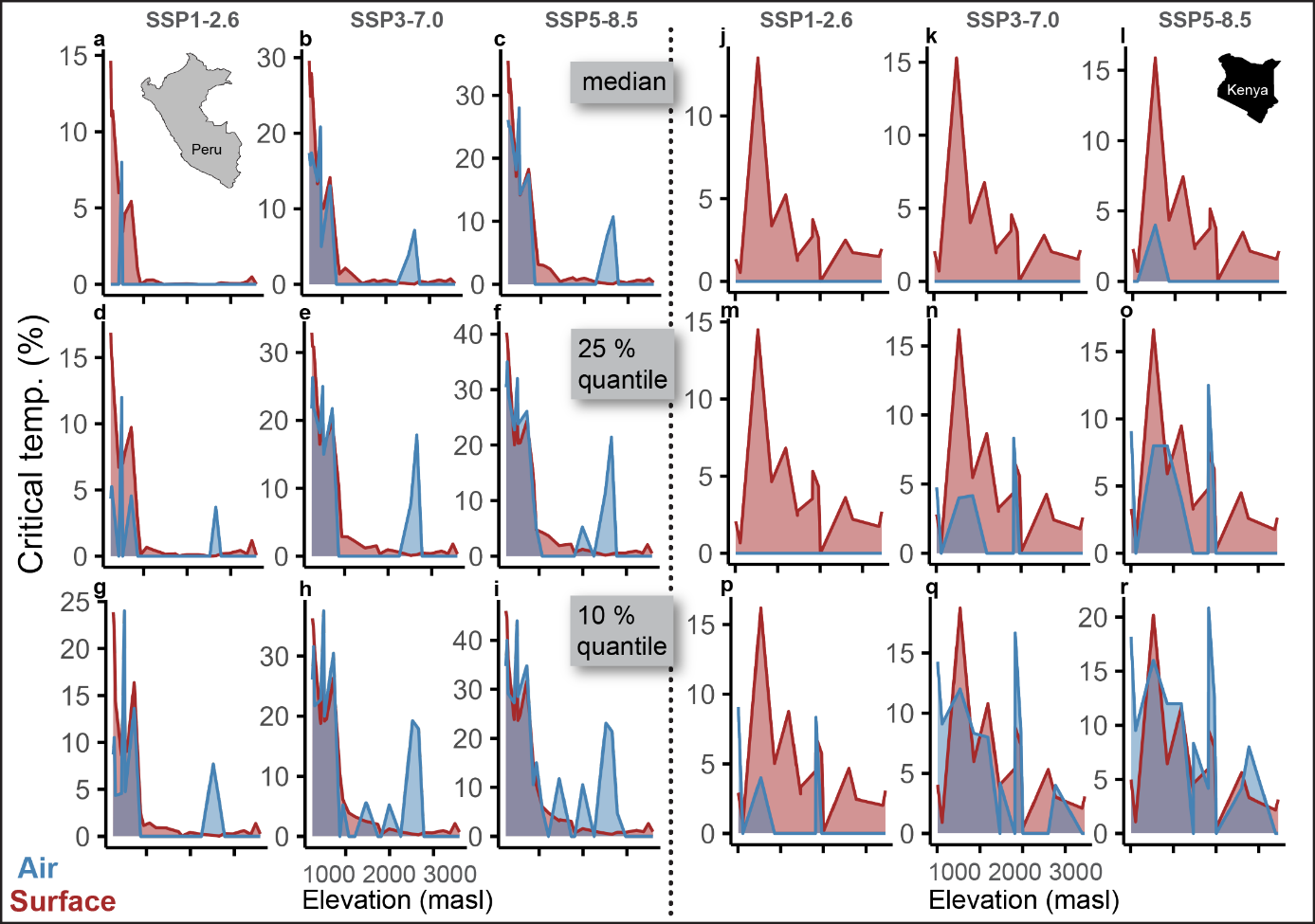
 **Figure S1 | Estimates of critical temperatures based on a multi-model ensemble.** Percentage of surface (red) and air (blue) future temperatures that are critical (i.e. can cause heat coma in less than 8 hours) at the Neotropical (Peru, left box, a–i) and East African (Kenya, right box, j–r) elevation gradient based on multi-model climate change anomalies (includes the models GFDL-ESM4, IPSL-CM6A-LR, MPI-ESM1-2-HR, MRI-ESM2-0 and UKESM1-0-LL; see Supplementary Methods); for median CT_max_ values (upper row), more heat-sensitive insects (25% quantile of CT_max_, middle row) and for the most heat-sensitive insects (10% quantile of CT_max_, lower row). The three shared socio-economic pathways SSP1-2.6, SSP3-7.0, and SSP5-8.5 are shown for each geographic region. Critical temperatures were calculated along the whole gradient assuming the CT_max_ from lowland organisms (Methods).

**Additional References**

Blaimer, B. B., B. F. Santos, A. Cruaud, M. W. Gates, R. R. Kula, I. Mikó, J.-Y. Rasplus, D. R. Smith, E. J. Talamas, S. G. Brady, and M. L. Buffington. 2023. Key innovations and the diversification of Hymenoptera. Nature Communications 14(1):1212. doi: 10.1038/s41467-023-36868-4

Cai, C., E. Tihelka, M. Giacomelli, J. F. Lawrence, A. Ślipiński, R. Kundrata, S. Yamamoto, M. K. Thayer, A. F. Newton, R. A. B. Leschen, M. L. Gimmel, L. Lü, M. S. Engel, P. Bouchard, D. Huang, D. Pisani, and P. C. J. Donoghue. 2022. Integrated phylogenomics and fossil data illuminate the evolution of beetles. Royal Society Open Science 9(3):211771. doi: doi:10.1098/rsos.211771

Cano, I. M., E. Shevliakova, S. Malyshev, J. G. John, Y. Yu, B. Smith, and S. W. Pacala. 2022. Abrupt loss and uncertain recovery from fires of Amazon forests under low climate mitigation scenarios. Proceedings of the National Academy of Sciences 119(52):e2203200119. doi: doi:10.1073/pnas.2203200119

IPCC. 2021. Climate Change 2021: The Physical Science Basis. Contribution of Working Group I to the Sixth Assessment Report of the Intergovernmental Panel on Climate Change, Cambridge, United Kingdom and New York, NY, USA.

Kawahara, A. Y., C. Storer, A. P. S. Carvalho, D. M. Plotkin, F. L. Condamine, M. P. Braga, E. A. Ellis, R. A. St Laurent, X. Li, V. Barve, L. Cai, C. Earl, P. B. Frandsen, H. L. Owens, W. A. Valencia-Montoya, K. Aduse-Poku, E. F. A. Toussaint, K. M. Dexter, T. Doleck, A. Markee, R. Messcher, Y. L. Nguyen, J. A. T. Badon, H. A. Benítez, M. F. Braby, P. A. C. Buenavente, W.-P. Chan, S. C. Collins, R. A. Rabideau Childers, E. Dankowicz, R. Eastwood, Z. F. Fric, R. J. Gott, J. P. W. Hall, W. Hallwachs, N. B. Hardy, R. L. H. Sipe, A. Heath, J. D. Hinolan, N. T. Homziak, Y.-F. Hsu, Y. Inayoshi, M. G. A. Itliong, D. H. Janzen, I. J. Kitching, K. Kunte, G. Lamas, M. J. Landis, E. A. Larsen, T. B. Larsen, J. V. Leong, V. Lukhtanov, C. A. Maier, J. I. Martinez, D. J. Martins, K. Maruyama, S. C. Maunsell, N. O. Mega, A. Monastyrskii, A. B. B. Morais, C. J. Müller, M. A. K. Naive, G. Nielsen, P. S. Padrón, D. Peggie, H. P. Romanowski, S. Sáfián, M. Saito, S. Schröder, V. Shirey, D. Soltis, P. Soltis, A. Sourakov, G. Talavera, R. Vila, P. Vlasanek, H. Wang, A. D. Warren, K. R. Willmott, M. Yago, W. Jetz, M. A. Jarzyna, J. W. Breinholt, M. Espeland, L. Ries, R. P. Guralnick, N. E. Pierce, and D. J. Lohman. 2023. A global phylogeny of butterflies reveals their evolutionary history, ancestral hosts and biogeographic origins. Nature Ecology & Evolution 7(6):903-913. doi: 10.1038/s41559-023-02041-9

Parry, I. M., P. D. Ritchie, and P. M. Cox. 2022. Evidence of localised Amazon rainforest dieback in CMIP6 models. Earth System Dynamics 13(4):1667-1675.

Song, H., O. Béthoux, S. Shin, A. Donath, H. Letsch, S. Liu, D. D. McKenna, G. Meng, B. Misof, L. Podsiadlowski, X. Zhou, B. Wipfler, and S. Simon. 2020. Phylogenomic analysis sheds light on the evolutionary pathways towards acoustic communication in Orthoptera. Nature Communications 11(1):4939. doi: 10.1038/s41467-020-18739-4

Song, N., M.-M. Wang, W.-C. Huang, Z.-Y. Wu, R. Shao, and X.-M. Yin. 2024. Phylogeny and evolution of hemipteran insects based on expanded genomic and transcriptomic data. BMC Biology 22(1):190. doi: 10.1186/s12915-024-01991-1
