# Supplementary material for: Limited thermal tolerance in tropical insects and its genomic signature
Source: Nature. 2026 Mar 4;651(8106):672–8. doi: 10.1038/s41586-026-10155-w (PMC12999521; doi:10.1038/s41586-026-10155-w)
Supplement: Supplementary file 2 — Reporting Summary [file 41586_2026_10155_MOESM2_ESM.pdf]

nature portfolio

Corresponding author(s): Kim L. Holzmann

Last updated by author(s): Jan 14, 2026

Reporting Summary

Nature Portfolio wishes to improve the reproducibility of the work that we publish. This form provides structure for consistency and transparency in reporting. For further information on Nature Portfolio policies, see our Editorial Policies and the Editorial Policy Checklist.

Statistics

For all statistical analyses, confirm that the following items are present in the figure legend, table legend, main text, or Methods section.

n/a	Confirmed	
☐	☒	The exact sample size (*n*) for each experimental group/condition, given as a discrete number and unit of measurement
☐	☒	A statement on whether measurements were taken from distinct samples or whether the same sample was measured repeatedly
☐	☒	The statistical test(s) used AND whether they are one- or two-sided<br>*Only common tests should be described solely by name; describe more complex techniques in the Methods section.*
☐	☒	A description of all covariates tested
☐	☒	A description of any assumptions or corrections, such as tests of normality and adjustment for multiple comparisons
☐	☒	A full description of the statistical parameters including central tendency (e.g. means) or other basic estimates (e.g. regression coefficient) AND variation (e.g. standard deviation) or associated estimates of uncertainty (e.g. confidence intervals)
☐	☒	For null hypothesis testing, the test statistic (e.g. *F*, *t*, *r*) with confidence intervals, effect sizes, degrees of freedom and *P* value noted<br>*Give P values as exact values whenever suitable.*
☒	☐	For Bayesian analysis, information on the choice of priors and Markov chain Monte Carlo settings
☒	☐	For hierarchical and complex designs, identification of the appropriate level for tests and full reporting of outcomes
☐	☒	Estimates of effect sizes (e.g. Cohen's *d*, Pearson's *r*), indicating how they were calculated

Our web collection on statistics for biologists contains articles on many of the points above.

Software and code

Policy information about availability of computer code

Data collection	N/A
Data analysis	All analyses were conducted in R (version 4.3) with the following packages: DECIPHER v2.30.0, ape v5.8, phylocomr v0.3.4, ips v0.0.12, phytools v2.3.0, ggtree v3.10.1, ggplot2 v3.5.1, ggnewscale v0.5.0, phylosignal v1.3.1, phylolm v2.6.5, geiger v2.0.11, phylolm.hp v0.0-3, NicheMapR v3.3.2, lme4 v1.1-36, DHARMa v0.4.7, officer v0.6.7, dplyr v1.1.4, tidyR v1.3.1, readxl v1.4.3, openxlsx v4.2.7.1. We used Anaconda PowerShell and Python (v. 3.13) to run DeepSTABp. The REfined Single Linkage alogorithm was used from BOLD version 4 (https://v4.boldsystems.org/). All code is available at https://doi.org/10.6084/m9.figshare.28891307.

For manuscripts utilizing custom algorithms or software that are central to the research but not yet described in published literature, software must be made available to editors and reviewers. We strongly encourage code deposition in a community repository (e.g. GitHub). See the Nature Portfolio guidelines for submitting code & software for further information.

Data

Policy information about availability of data

All manuscripts must include a data availability statement. This statement should provide the following information, where applicable:

- Accession codes, unique identifiers, or web links for publicly available datasets
- A description of any restrictions on data availability
- For clinical datasets or third party data, please ensure that the statement adheres to our policy

All field-collected data is publicly available in the FigShare repository (https://doi.org/10.6084/m9.figshare.28891307). Climate data from CHELSA is publicly available at https://www.chelsa-climate.org/ (BIOCLIM+ data set). ECOSTRESS surface temperatures are available from NASA Earthdata (https://doi.org/10.5067/ECOSTRESS/ECO_L2G_LSTE.002 [29]). Insect genomes are from InsectBase 2.0 (https://v2.insect-genome.com/).

Research involving human participants, their data, or biological material

Policy information about studies with human participants or human data. See also policy information about sex, gender (identity/presentation), and sexual orientation and race, ethnicity and racism.

Reporting on sex and gender	N/A
Reporting on race, ethnicity, or other socially relevant groupings	N/A
Population characteristics	N/A
Recruitment	N/A
Ethics oversight	N/A

Note that full information on the approval of the study protocol must also be provided in the manuscript.

Field-specific reporting

Please select the one below that is the best fit for your research. If you are not sure, read the appropriate sections before making your selection.

☐ Life sciences ☐ Behavioural & social sciences ☒ Ecological, evolutionary & environmental sciences

For a reference copy of the document with all sections, see nature.com/documents/nr-reporting-summary-flat.pdf

Ecological, evolutionary & environmental sciences study design

All studies must disclose on these points even when the disclosure is negative.

Study description	In the study we tested the effects of climate (mean annual temperature, quantitative variable) and phylogenetic relations on thermal limits of tropical insects along two elevation gradients in the Andean-Amazonian ecosystem (Peru) and in East Africa (Kenya). We tested the underlying mechanism of physiological thermal limits by investigating insect protein melting temperatures. For most response variables the sample size was 26 in the Neotropics and 15 in the Afrotropics; for few response variables the sample size was lower as data was missing for some study sites. The sample size is indicated for each response variable in the method section. As all study sites were geographically seperated by an elevational interval of 250 m and a linear distance of at least 400 m, and on average all study site pairs were seperated by 1.5 km, we considered study sites as independent replicates and used gernalized additive or linear models (e.g. linear regression) for nearly all of the analyses.
Research sample	We tested thermal limits of insect species across six major orders: Diptera, Coleoptera, Hymenoptera, Hemiptera, Lepidoptera, Orthoptera.
Sampling strategy	The number of study sites (26 in the Neotropics, 15 in the Afrotropics) represents a balance between good statistical power and feasability of field work and was chosen based on the great experience of many included authors in the performance of tropical field work and the analyses of ecological data sets.
Data collection	Several of the authors (KLH, TS, AA, MC) collected the data in the field.
Timing and spatial scale	Data was collected from September 2022 to December 2023 with a gap (January 2023 to April 2023) during the strong rain season in the Neotropics, since roads are often not accessible and field work would be not feasible during this time.
Data exclusions	No data was excluded.
Reproducibility	We used a common protocol to test thermal limits of insects and measured ~8000 individuals. All details are provided in the

Reproducibility	methods, so that our protocol can be repeated. In both geographic regions we applied the same standardized measurements so that data can be compared. We controlled for differences in taxonomy by using DNA sequences from each tested insect to create a phylogenetic tree.
Randomization	Insect samples were randomly collected at each plot and randomly assigned to be tested either for their upper or lower thermal limit.
Blinding	Blinding is not relevant to our study, since we analysed a real mountain ecosystem by standardised methods, that creates a numerical outcome which is not subject to personal interpretation.

Did the study involve field work? ☒ Yes ☐ No

Field work, collection and transport

Field conditions	In Peru, the study was carried out along an elevational gradient from 245 meters above sea level (masl) to the tree line at 3588 masl in the Andes of south-east Peru (Kosñipata valley), with continuous and mostly undisturbed wet rainforest/cloud forest. Mean annual temperatures range from 24.3 °C in the lowlands to 6.7 °C at 3600 masl. Mean annual precipitation levels are high with > 1500 mm per year along the whole gradient, peaking at around 1500 masl with ~5000 mm. In Kenya, the study was carried out along an elevational gradient from 11 masl at Watamu to 3450 masl at Mount Kenya including forests, woodland, scrub, and grassland in natural and semi-natural habitats. Mean annual temperatures range from 26.2 °C at the lowest plot to 8.9 °C at the highest plot. The forested parts of the Taita Hills region and Mount Kenya from ~1300–2500 masl are characterized by a tropical montane forest climate with generally high humidity and constant high precipitation (> 1500 mm).
Location	In Peru, the gradient ranged from -12,57079 -70,09245 at an elevation of 245 masl to -13,09608 -71,62952 at an elevation of 3588 masl. In Kenya, from -3,37667 39,98829 at an elevation of 11 masl to -3,37667 39,98829 at an elevation of 3450 masl.
Access & import/export	All data collection were conducted under appropriate permits. In Peru, SERNANP provided access to Manu National Park (N° 18-2022-SERNANP - JEF), and ERFOR provided research permits (Nº D001044-2022-MIDAGRI-SERFOR-DGGSPFFS-DGSPFS). In Kenya, we had permits from the JRS Biodiversity Foundation (grant No: 60930) and in fieldwork was authorized by NACOSTI under License No: NACOSTI/P/22/20735. This study was accomplished within the scope of the Research Unit ANDIV (www.andiv.biozentrum.uni-wuerzburg.de) and funded by the Deutsche Forschungsgemeinschaft (DFG) under grant PE 1781/4-1.
Disturbance	We did not experimentally modify the study plots. Insects had to be collected, killed and preserved in order to conduct the study; however, we are confident that this has no impact on the species populations.

Reporting for specific materials, systems and methods

We require information from authors about some types of materials, experimental systems and methods used in many studies. Here, indicate whether each material, system or method listed is relevant to your study. If you are not sure if a list item applies to your research, read the appropriate section before selecting a response.

Materials & experimental systems

n/a	Involved in the study	
☒	☐	Antibodies
☒	☐	Eukaryotic cell lines
☒	☐	Palaeontology and archaeology
☐	☒	Animals and other organisms
☒	☐	Clinical data
☒	☐	Dual use research of concern
☒	☐	Plants

Methods

n/a	Involved in the study	
☒	☐	ChIP-seq
☒	☐	Flow cytometry
☒	☐	MRI-based neuroimaging

Animals and other research organisms

Policy information about studies involving animals; ARRIVE guidelines recommended for reporting animal research, and Sex and Gender in Research

Laboratory animals	No laboratory animals were used.
Wild animals	During the study we collected 7994 insects from six orders (Diptera, Hymenoptera, Coleoptera, Lepidoptera, Orthoptera), comprising 2330 unique genetic units, no morphological identification on species level was made and no information on age, strain or sex were collected. The specimen are preserved and will be stored in museum collections in Peru and Kenya.
Reporting on sex	No information on sex has been collected.
Field-collected samples	Samples collected in the field were transported back to the station in 2-50 ml tubes equipped with sugar water within two hours,

Field-collected samples	maintained at the ambient temperature of the collection site, no exposure to light, and experiments were conducted the same day. Afterwards samples were stored in ethanol.
Ethics oversight	No ethical approval was required since lower invertebrates (insects) were tested.

Note that full information on the approval of the study protocol must also be provided in the manuscript.

Plants

Seed stocks	*Report on the source of all seed stocks or other plant material used. If applicable, state the seed stock centre and catalogue number. If plant specimens were collected from the field, describe the collection location, date and sampling procedures.*
Novel plant genotypes	*Describe the methods by which all novel plant genotypes were produced. This includes those generated by transgenic approaches, gene editing, chemical/radiation-based mutagenesis and hybridization. For transgenic lines, describe the transformation method, the number of independent lines analyzed and the generation upon which experiments were performed. For gene-edited lines, describe the editor used, the endogenous sequence targeted for editing, the targeting guide RNA sequence (if applicable) and how the editor was applied.*
Authentication	*Describe any authentication procedures for each seed stock used or novel genotype generated. Describe any experiments used to assess the effect of a mutation and, where applicable, how potential secondary effects (e.g. second site T-DNA insertions, mosiacism, off-target gene editing) were examined.*
